# Frontier injustice: the American threat to Canada’s drug supply

**DOI:** 10.17269/s41997-019-00238-9

**Published:** 2019-07-08

**Authors:** Adam R. Houston, Amir Attaran

**Affiliations:** 1 Faculty of Law, University of Ottawa, 1 Stewart St, Room 221, Ottawa, ON K1N 6N5 Canada; 2 Faculties of Medicine and Law, University of Ottawa, 1 Stewart St, Room 221, Ottawa, ON K1N 6N5 Canada

**Keywords:** Canada, United States, Drug importation, Drug pricing, Drug shortage, Canada, États-Unis, Importation de médicaments, Prix des médicaments, Pénurie de médicaments

## Abstract

“Import-from-Canada” strategies to address high prices for patented pharmaceuticals are gaining momentum in the United States. In the first two months of 2019 alone, five congressional bills incorporating such strategies were introduced; these bills have attracted bipartisan support across the fractious American political divide. At the same time, Canadian lawmakers continue their long-standing failure to take action in response to the looming threat of having Canada’s drug supply siphoned off by its more powerful neighbour. Unregulated bulk importation of pharmaceuticals originally intended for the Canadian market into the USA would lead to severe drug shortages that would undermine the Canadian health system, while also halting emerging moves towards universal Canadian pharmacare in their tracks. At the same time, this short-term approach to tackling American pricing woes would fail to heal the deep systemic issues that underpin unaffordable drug prices in the USA. This article underscores the need for Canada to take action to protect its supply of patented medicines, and suggests possible forms such action might take.

## Résumé

Les stratégies d’« importation du Canada » pour faire face aux prix élevés des produits pharmaceutiques brevetés prennent de l’ampleur aux États-Unis. Dans les deux premiers mois de 2019, cinq projets de loi du Congrès incorporant de telles stratégies ont été présentés; ces projets de loi ont attiré un soutien bipartite à travers le clivage politique hargneux des États-Unis. Parallèlement, les législateurs canadiens maintiennent leur inaction de longue date face à la menace imminente de voir l’approvisionnement des médicaments au Canada siphonné par son voisin plus puissant. L’importation en vrac non réglementée de produits pharmaceutiques initialement destinés au marché canadien aux États-Unis entraînerait de graves pénuries de médicaments qui compromettraient le système de santé canadien, tout en freinant l’évolution en cours vers un régime universel d’assurance-médicaments au Canada. En même temps, cette approche à court terme visant à résoudre les problèmes de tarification des États-Unis ne parviendrait pas à adresser les problèmes systémiques profonds qui sous-tendent les prix inabordables des médicaments aux États-Unis. Cet article souligne la nécessité pour le Canada de prendre des mesures pour protéger son approvisionnement en médicaments brevetés et suggère des formes possibles que pourraient revêtir de telles actions.

Even as exaggerated tales of illicit drugs flooding over the border are used by American politicians to support calls to build a wall between Mexico and the United States, the very real threat posed by the unregulated flow of prescription drugs into the USA from Canada has been almost completely overlooked by their Canadian counterparts. A beggar-thy-neighbour approach to resolving American pharmaceutical pricing woes through mass importation of cheaper drugs from their northern neighbour would be disastrous for Canadians.

So-called parallel importation of patented drugs from Canada, as an alternative to the identical product sold at a higher price in the USA, is one of the few issues that a majority of Americans, Republican or Democrat, can agree on (Kirzinger et al. 2017). The idea is not new; the U.S. Congress passed the underlying legal framework to permit wholesale importation from Canada in 2003 (Importation of Prescription Drugs – U.S. Code 2003). To take effect, new state laws implementing importation only require the approval of the federal Secretary of Health & Human Services. While approval has progressed haltingly under the Trump administration, it could change quickly if, as seems likely, parallel importation turns into a bellwether—or outcome—of the 2020 election cycle.

Within the first two months of the current 2019–2020 U.S. Congressional session, five bills were introduced to permit importation of drugs from Canada, whether for personal use or wholesale. As the U.S. Congressional website shows, more than one of these bills was introduced with bipartisan support from Republicans and Democrats, while numerous candidates for the Democratic presidential nomination have sponsored at least one. Meanwhile, at the state level, the idea is being debated from California to Utah, with advocates proffering model legislation for importation. In 2018, Vermont became the first state to enact such a law (Roubein and Seipel 2018).

The American perspective, unsurprisingly, focuses on the prospect of access to cheaper medicines as a boon to Americans struggling to afford their prescriptions. It is also shaped by the phenomenal power of the pharmaceutical lobby, which throws more lobbying dollars around Washington than many other influential industries combined. That power has defeated nearly all attempts at restraining domestic drug prices, to the point government programs like Medicare are forbidden from negotiating prices.

Faced with an intolerable situation, and no clear path to systemic reform, Americans find the alternative of parasitizing Canada’s more affordably priced supply of patented drugs attractive, particularly since the foundational federal legislation necessary to do so is already in place. Advocates point to the fact that the same drug, sold by the same pharmaceutical company, can cost four times as much, or more, in the USA as it does in Canada (Prescription 2018). The difference occurs because Canada’s government regulates the prices of patented medicines through its Patented Medicine Prices Review Board (PMPRB), which ensures that prices in Canada are near the median of similar peer countries, most of which also regulate prices. Ironically, Canada’s government has been considering dropping the USA as a peer country in this calculation precisely because it does not have measures in place to protect consumers from high drug prices (Regulations 2017).

There is no doubt that parallel importation would save Americans money. The Congressional Budget Office estimated that an earlier proposal by Senator Bernie Sanders would save $6.8 billion over 10 years (Silverman 2017). Private providers would save money too; a limited evaluation of the Vermont law focusing on 17 of the most widely used drugs concluded that even with 45% markup on Canadian drugs, participating commercial payers stood to save millions of dollars annually (Backus 2018).

But where America seeks a green light, Canada needs to see a red flag. A large-scale transfer of Canada’s drug supply to its neighbour with ten times the population would have drastic, potentially devastating effects on Canada’s health system. A 2010 study on the potential effects of US importation concluded that “if 10% of the U.S. prescriptions were filled from Canadian sources (manufacturer, wholesale or retail), Canada’s 2007 drug supply would be exhausted in 224 days” (Shepherd 2010). Prices, while mitigated by the PMPRB, would rise. Worse, drug manufacturers would have no incentive to replenish Canada’s supply, knowing that the drugs would simply be redistributed to America’s larger and more lucrative market, thereby sapping their profits there (Rawson and Binder 2017). Quite likely, drug makers would go further and withhold supplies to Canada in the first place. This is precisely what happened in the early 2000s, when the pharmaceutical company GlaxoSmithKline (GSK) cut off supplies to a number of Canadian internet pharmacies in order to prevent them from undercutting the American market (Zehr 2003). State-sanctioned bulk importation would implicate far higher volumes than online sales to individual patients did. In either scenario—failing to replenish supplies, or outright withholding them—drug shortages in Canada would be guaranteed.

These adverse consequences of American zeal for parallel importation are little acknowledged. Even American clinicians, who presumably have some commitment to medical ethics, seem largely unconcerned about—or simply oblivious to—the fact that supplying an American patient in this way could mean taking that same drug away from a Canadian patient (Attaran et al. 2016). Certainly, none of the US politicians championing parallel importation seems to have hesitated over the distributive injustice of a “solution” to the US pricing problem that is tantamount to raiding the medicine chest of America’s closest ally.

But the real travesty lies with Canada’s government, which, over the nearly two decades that America has pondered this option, has utterly failed to take action to protect its citizens. This has been clear since 2003, when the Canadian Competition Bureau declined to take any action against GSK over restricting the supply of their products (Elliott 2003). That decision stands as precedent that pharmaceutical companies can close the taps to Canada without repercussions.

No contemporary Canadian politician has commented on what Canada would do if, as now seems probable, the US implements parallel importation. The closest step towards decisive action to protect Canada’s drug supply was taken in 2005, when federal Minister of Health Ujjal Dosanjh declared that “Canada cannot be the drugstore for the United States of America”, and indicated he was bringing forward legislation to restrict the export of Canadian drugs to the USA (Canada 2005). No such legislation was passed, however, before the Liberal government fell in an election. In 2007, a Private Member’s Bill to similar effect was introduced in Parliament, but was voted down by the right-leaning Conservative Party and left-leaning New Democratic Party (Bennett 2006). Several years later, the former Conservative Health Minister Leona Aglukkaq seemingly had second thoughts, and published an editorial in the *Washington Post* warning about parallel importation*,* but neither she nor her Cabinet colleagues took steps to mitigate the risk while in power (Aglukkaq 2017).

The Canadian government’s lack of concern is highly alarming. Without a legal framework in place to prevent large-scale export of even the most essential, life-or-death drugs to America, there is no reason that US medicine traders could not pay Canadian wholesalers a modest percentage over the going Canadian price, so as to arbitrage most (or even all) of a drug’s supply across the border. Given the bipartisan popularity of “import from Canada” proposals at federal and state levels in America, Ottawa’s continued failure to consider—much less enact—protective legislation for Canada’s drug supply lacks rational explanation.

There are a number of legislative steps Canada could take to protect itself. For one, Canada could unconditionally prohibit the bulk export of pharmaceuticals that are packaged and labelled for the Canadian market, whatever their origin. This could be done without affecting the legitimate export of medicines manufactured by Canadian pharmaceutical firms specifically for foreign markets.

Alternatively, Canada could install a regulated system requiring all those exercising American parallel importation laws to also obtain licensure as parallel exporters in Canada. This would allow Canada to require export permits for each US-bound shipment, and to recapture something of the price gap between Canadian and American prices through licensing and permitting fees, while also keeping a throttle hand on the export of drugs to avoid critical shortages at home. Both these options are consistent with trade agreements such as the *North American Free Trade Agreement* and its recently negotiated successor, which allow for exceptions from free trade where necessary to protect human or animal health.

While Canada has a number of options to choose from, continued inaction is not one of them. Without legal defences of its own, Canada faces the impending risk of having its drug supply siphoned off by a more powerful, increasingly desperate neighbour. Not only would this threaten Canadian lives, it presents a strategic hurdle to the Canadian government’s pending plans to introduce universal pharmacare. It would also hurt the USA by putting off the inevitable need to tame the excesses of its pharmaceutical lobby, which stands in the way of other reforms Washington needs to enact. Canada’s continued political lethargy constitutes borderline negligence.
